# Statistical-based detection of pilot contamination attack for NOMA in 5G networks

**DOI:** 10.1038/s41598-025-86020-z

**Published:** 2025-01-29

**Authors:** Dalia Nashat, Sahar Khairy

**Affiliations:** Department of Information Technology, Faculty of Computers and Information, Assiut University, Assiut, Assiut 71515 Egypt

**Keywords:** Fifth generation (5G), Millimeter wave (mmWave), Massive multiple-input-multiple-output (MIMO), Non-orthogonal-multiple-access (NOMA), Pilot contamination attack (PCA), Channel state information (CSI), Electrical and electronic engineering, Energy infrastructure

## Abstract

Fifth-generation (5G) communication technologies, such as millimeter wave communication, massive multiple-input-multiple-output and non-orthogonal-multiple-access (NOMA) are playing a pivotal role in promoting the modern applications of the Internet-of-Things. Using non-orthogonal resource allocation, NOMA can increase spectrum efficiency and achieve wide connectivity with low transmission delay and signaling cost. Despite the high potential of NOMA in 5G communications, NOMA is susceptible to a pilot contamination attack (PCA), in which an attacker resents the same pilot signals as authorized users. Currently, using the available detection methods in NOMA gives high false positive probability since the time-division-duplex or orthogonal resource block can be allocated by many authorized user. Since the pilot contamination attack changes the signal reception at the legitimate receiver, this work introduces a novel detection scheme for identifying Pilot Contamination attack (PCA) that statistically investigates the asymmetry in received signal power levels. The main idea of the proposed detection scheme is to use various statistical measurements for normal traffic attributes (CSI) as a reference profile. Then, compute the Mahalanobis distance between the reference profile and CSI for the incoming connection and use the probability of the uniform distribution to make the final detection decision. The performance of the proposed detection technique in terms of its detection rate and false positive probabilities has been evaluated through extensive simulation. The simulation results show that the proposed scheme succeeded in detecting the pilot contamination attack with a detection rate of up to 98% and a precision reached 97.88%.

## Introduction

Recently the applications of IoT have been used in almost every aspect of modern life. Fifth-generation (5G) communication technologies, such as millimeter wave communication (mmWave), massive multiple-input multiple-output (MIMO) and non-orthogonal multiple-access (NOMA) are playing a key role in promoting these applications. More and more heterogeneous devices with unknown security setup have joined 5G wireless networks. Therefore, these devices can be compromised to become botnets.

Modern wireless networks use two main multiple-access techniques, orthogonal multiple access (OMA) and non-orthogonal multiple access (NOMA). OMA is one of the earlier used techniques in wireless network generations that includes frequency division multiple access (FDMA) in the first generation (1G), time division multiple access (TDMA) in the second generation (2G), code division multiple access (CDMA) in the third generation (3G) and orthogonal frequency division multiple access (OFDMA) in the fourth generation (4G)^1^. OMA uses orthogonal resource ( frequency, time and code ) allocation for all users, this means that each user is allocated one block and this block can not be used by another user. Since there is no reciprocal interference among users in an ideal scenario, OMA approaches can achieve good performance even with simple receivers. It is notable, however, that in the current cellular wireless networks, the number of users increases rapidly due to widespread mobile devices. Therefore, OMA techniques may not meet the stringent emerging requirements such as extremely high spectral efficiency, very low latency and massive device connectivity.

The special characteristics in 5G wireless communications provide Physical Layer Security (PLS) new opportunities to counter physical-layer threats in IoT systems. For example, beamforming in massive MIMO can be used to reduce the risk of eavesdropping. Physical Layer Security (PLS) has new chances to combat physical-layer attacks in IoT systems because of the special characteristics of 5G wireless connectivity. In mmWave MIMO networks, high propagation loss and directionality can be deployed to prevent spoofing and eavesdropping^2^ and the unique communication properties used to detect pilot contamination attack^3,4^. Moreover, band full-duplex ability in 5G IoT devices can block jamming attacks^5,6^. On the other hand, technologies for 5G open the door for physical-layer threats in IoT networks.

NOMA is one of the most critical enabling technologies for 5G wireless networks^7^. It has been developed as a solution for increasing spectral efficiency while allowing some multiple access interference at receivers. Contrary to OMA, NOMA uses non-orthogonal resource allocation for users and can allocate one block for many users. Moreover, NOMA can communicate with all authorized users in the same block with accurate Channel State Information (CSI). Also, NOMA can accomplish broad connections with low transmission latency and minimum signaling expense.

**Fig. 1:**
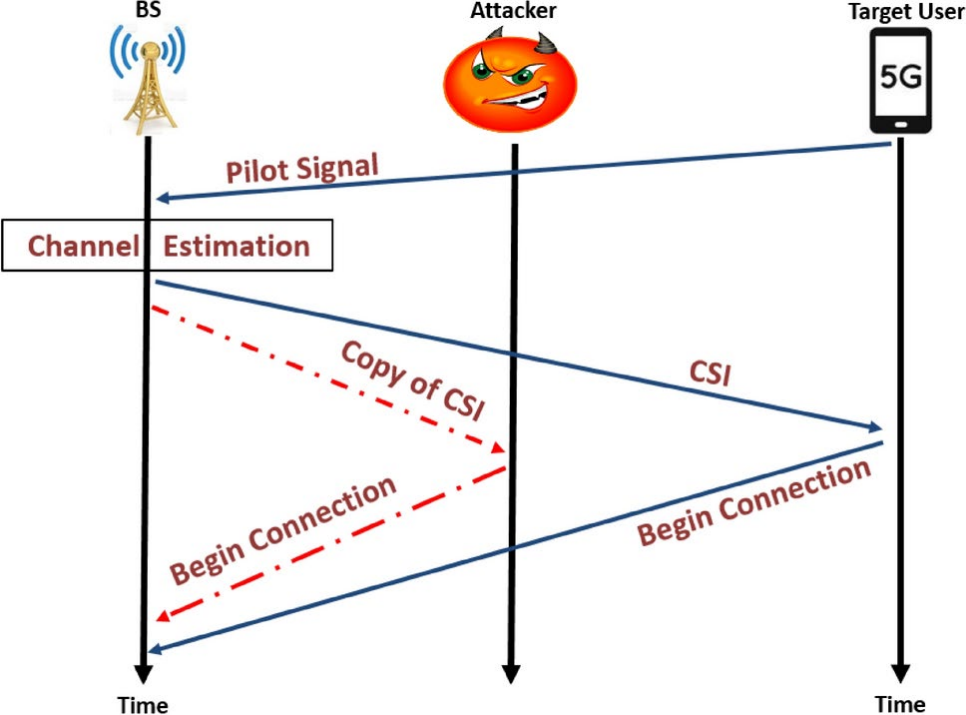
Pilot-contamination attacks on the uplink.

**Fig. 2:**
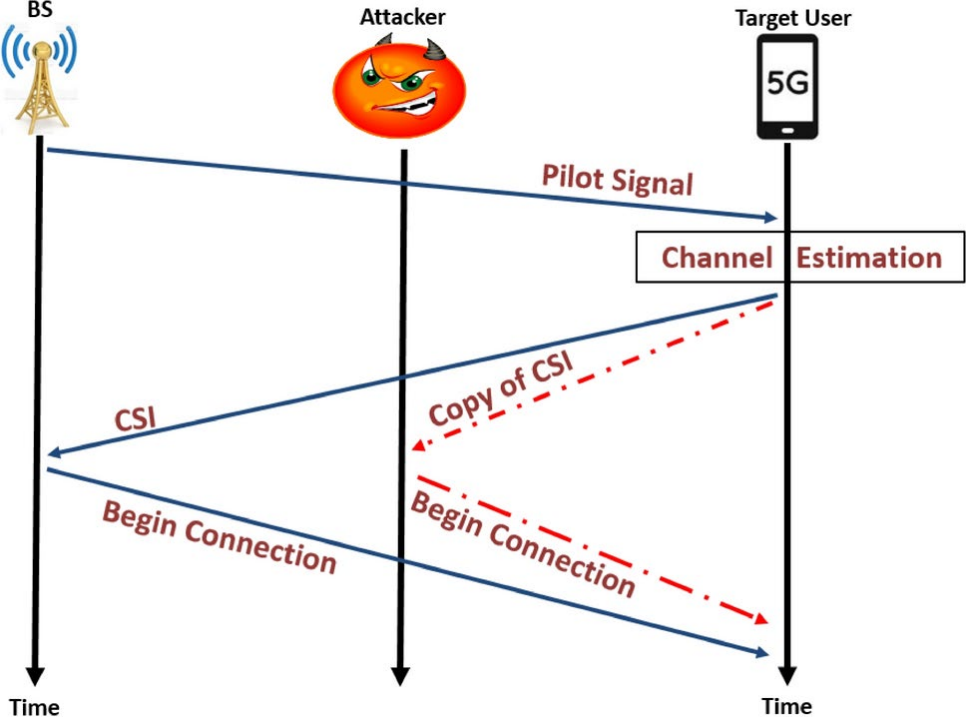
Pilot-contamination attacks on the downlink.

NOMA is vulnerable to the pilot-contamination attack (PCA) in uplink and downlink as shown in Figs. 1 and 2 respectively, which allows an attacker to send the same pilot signals as an authorized user. The PCA attacker can inject the identical pilot signal during the channel training phase like the authorized users to control the channel estimate outcome and influence the precoding procedure. The attacker not only can interfere with the legitimate receiver’s ability to receive the signal but also allow the communication system to leak private data.

Detecting pilot contamination attacks is more difficult in 5G wireless networks which deploy NOMA techniques than OMA because NOMA can allocate one block for many users. This allocation way leads to superimposed and convoluted communication signals. Moreover, active attackers have an advantage for launching jamming or eavesdropping attack if they have full-duplex capability.

Most existing detection methods for PCA depend on the concept of orthogonality (i.e., the pilot signals of multiple users are assigned to different orthogonal resource blocks), so they can distinguish between legitimate and attack pilot signals. Therefore, more than a pilot signal in the same block can be detected as a pilot contamination signal, and this increases the false positive probability in NOMA technique. This work presents a novel detection method that can detect PCA when the pilot signals of multiple users are assigned to non orthogonal resource blocks.

Based on the fact that the pilot contamination attack can reduce the signal reception at the legitimate receiver in the downlink and increase the signal reception at the legitimate receiver in the uplink, the available detection schemes for PCA compared current Channel State Information (CSI) with previous CSI to detect the contamination attack. In order to investigate the asymmetry of received signal power levels at the legitimate receiver, we suggest statistically-based detection scheme for pilot contamination attacks. The main idea of this proposed detection scheme is to use various statistical measurements for normal traffic attributes (CSI) as a reference profile. Then, we compute the Mahalanobis distance between the reference profile and CSI for the incoming connection and use the probability of the uniform distribution to make the final detection decision.

The rest of this paper is structured as follows. Section “Related work” introduce related works. Section “Pilot contamination attack” describe PCA model, challenges and motivation. The proposed detection scheme introduced in Section “The proposed detection scheme”. Section “Performance evaluation evaluate the performance and finally, Section Conclusion concludes this paper.

## Related work

Superimposed and complex broadcast signals of NOMA make PCA hard to detect or defend. Moreover, the attacker follows the legitimate user’s behavior to decrease the detection probability. The concept behind most available detection techniques is investigating the asymmetry of received signal power levels at legitimate users.

The author in^8^ used a deep-learning model, namely Generative Adversarial Networks (GAN) which consists of two independent neural networks, namely the Discriminator (D) and the Generator (G) as^9^. The training phase is conducted in Python, using Googleś Colab with 5 different threshold values ( 0.001, 0.002, 0.003, 0.004, 0.005 ) and 4000 data set used as the input of GAN. The average of results which have achieved in five cases are 92 Accuracy, 85.66 Recall, 90.9 F1 Scoring and 100 Precision. Using a fixed threshold usually based on statistics of the connection attributes. it is notable, however, that the number of attributes in every time vary dramatically according to the users activity and mobility. GAN scheme detect the abnormal PCA traffic behavior, but the main disadvantage of GNA that it need a large amount of traffic data for training which is not practically applicable.

By examining the asymmetry of received signal power levels at the transmitter and the legitimate receiver, Xiong Qi et al.^10^ presented an energy ratio detector (ERD) for detecting PCA. Their detection process primarily consists of two steps: first, the legitimate receiver (Bob) transmits the assigned pilot signal to the transmitter (Alice) via the uplink channel, and Alice estimates the channel based on samples of signals. Second, Alice calculates the received signal power, modulates that as a data signal and broadcasts it via a downlink channel. Bob calculates the strength of his received signal after demodulating the data. Then it compares the two power levels to determine whether there is an attack. However the ERD detector has outstanding detection performance, it did not suggest any clear backup procedures to restore secure data transport. Hence, to accomplish the objectives of detecting the pilot spoofing attack and securely re-transmitting the data signal, the same authors provided a two-way training-based approach in^11^

A pilot contamination detection method is proposed in^7^. This method can be used in wireless IoT systems with NOMA communication scenarios. It uses signal processing at the access point, necessitating no further signal design or modification at the legitimate user (i.e., IoT device). Two efficient detection algorithms are deployed for static and dynamic environments by using the sparsity and statistics of mm-Wave and massive MIMO virtual channels.

Analysis and experimentation were used to explore the effects of jamming during channel sounding in real-world Massive MIMO networks by Zhang et al.^12^. They present and model the Pilot Distortion Attack (PDA) which is a straight forward but effective jamming technique that can cause denial-of-service of all clients connected to the AP. They proposed jamming attack detection system by estimating the multiple-antenna carrier frequency offset with zero startup and no additional network cost.

The authors in^13^ adopted the perspective of the eavesdroppers and looked at the possibility of many eavesdroppers working together to design the Pilot Spoofing Attack to improve wiretapping performance. They assume that the Eves are aware of their own CSI, allowing them to modify and improve their attacking signals accordingly. They give a more thorough assessment of the potential secrecy issues in wireless communication networks by taking into account multiple Eves.

To identify users’ symbols in the cooperative NOMA system, the authors in^14^ presented a semi-blind mutual detection scheme-based on Deep Learning. Since the suggested technique could achieve a simultaneous detection based on pilot responses, it was able to identify the signal without needing for further channel estimate procedures. The trained network had been used in the online detection phase after the Deep Learning model had been trained offline over a Rayleigh fading channel. Finally, the trained model further tested utilizing Rician and Na-agami-m fading channels.

Authors in^15^ merged SIC detection structure with a deep learning model to decrease the learnable parameters. On the uplink single input multi output (SIMO) NOMA system, they presented a receiver structure with a combined channel estimate and signal identification approach for random channels. At the SIC detection step, adding noise and interference mitigation settings increased signal identification precision and decreased noise interference.

An Orthogonal Frequency Division Multiplexing-NOMA (OFDM-NOMA) system with simultaneous channel estimation and signal detection based on Deep Learning has been proposed in^16^. Both signal identification and channel estimation are done concurrently. Using the data and pilot signals they have received, users can identify their own signals.

A detection method for pilot spoofing attack in massive MIMO systems based on special signals sent from the BS to authorized users has been proposed in^17^. Their approach calculates the large-scale fading factors instead of assuming theme as a priori information.

Channel virtual representation was used to detect pilot contamination attacks in mmWave MIMO communications in^18^. Their scheme was intended to highlight properties of mmWave communication channels. Due to the strong directivity of mmWave communications, the multipath channel features resulting from reflection and refraction are sensitive to transmitter position.

A likelihood ratio method has been proposed in^19^ to detect the pilot spoofing attack for a three-terminal massive mmWave MIMO system with a cooperative relay. After the received pilot signal has been forwarded from the cooperative relay, the equivalent CSI from the base station to the user was obtained and the joint CSI from the user/attacker to the relay was derived. Finally, the likelihood ratio was calculated to detect the pilot spoofing attack.

We introduce a comparison between some detection methods in Table 1

**Table 1:** Comparison between some detection methods.

References	Method	Advantages	Disadvantages
^10^	Eenergy ratio detector (ERD)	No need for prior information	Haigh false alarm if user near antennae
^11^	Using two-way training-based approach	Secure in re-transmitting the data signal	Need Large training data
^12^	Estimating carrier frequency offset for multiple anntena	No additional network cost	Can cause a DoS attack at AP.
^7^	Using signal processing	No modification at the legitimate user	May lead to a false alarm.
^18^	Using channel virtual representation	High detection rate	High false positive.
^8^	Using a deep-learning model (GAN)	High detection rate	Need large training data

##  Pilot contamination attack

Compared with traditional Internet of Things (IoT) networks, 5G IoT networks have a wider range of applications, including Internet of Vehicles (IoV), smart healthcare, smart homes, smart cities, Unmanned Aerial Vehicle (UAVs) and Industry 4.0^20^. Technologies for 5G wireless communication increase the possibility for physical-layer threats in the IoT networks since some new 5G wireless technologies may be more vulnerable to current physical layer attacks. For instance, massive MIMO and NOMA communications are more sensitive to pilot contamination attacks.

Attacks for physical layer in 5G networks can be classified to four categories based on the main target of attackers (i.e. Eavesdropping, Contaminating, Spoofing, and Jamming) as follows:

*Eavesdropping:* attackers listening in certain sensitive information by interception. Since the attackers don’t sends any signals, it is hard for legitimate transmitters or receivers to identify or track down the eavesdroppers identity. Based on the attacker behaviour, there are two types of this physical-layer threat: traffic analysis and interception^21,22^.

*Spoofing:* attackers try to join or disrupt legal communications by inserting some falsified identity information. There are two common types Identity spoofing and Sybil attacks^20^.

*Jamming:* a jamming attacker aims to block legitimate communications by making noise^23^. The attacker could do this by continuously transmitting radio signals on a wireless channel to obstruct communication by lowering the signal-to-noise ratio (SNR). At the physical layer, this can lead to denial-of-service attacks^24^. The three main types of jamming attacks are pilot jamming, proactive jamming, and reactive jamming.

*Contaminating:* attackers want to pollute the channel estimate phase in order to gain illegitimate advantages in the following communication phase^25^. This type of attack can be divided into pilot and feedback contamination.

In the present work, we will focus on the pilot contamination attack as one example of an active eavesdropping activity carried out by a malicious user during the channel training phase. This attack can affect the outcome channel estimation by transmitting pilot (training) signals which are identical to those of the legal users. This may lead to an increased channel rate for the attacker but a decreased channel rate for the legitimate receiver^11^. Additionally, PCA is one of the main performance bottlenecks that prevent massive MIMO from reaching its full potential.

According to different channel estimation stages, this attack can be divided into two types, pilot contamination attack on the uplink and the downlink. In *pilot contamination on the uplink*, the legitimate user begins the connection to BS or AP by sending a pilot signal and the BS estimates the CSI and retransmits it to all users. In this stage, the attacker copies the CSI of a legitimate user and uses it to communicate with BS as shown in Fig. 1. In the other hand in *pilot contamination on the downlink* the BS begins the connection to its users through broadcasting a pilot signal, then the legitimate user estimates the CSI and retransmits it to BS. Thus, the attacker copies the CSI for a legitimate user and uses it to communicate with BS^11,20^ as illustrated in Fig. 2. In both instances, the AP or BS will estimate the CSI between itself and the legitimate users incorrectly. This yield to incorrect precoding, beamforming or successive interference cancellation (SIC) at the AP or BS^20^.

As mentioned before, PAC results from the huge MIMO network’s reuse of pilot sequences. Consequently, the pilot sequences allocated to various network users are not orthogonal^26,27^. Researchers emphasized the need for next-generation wireless networks to handle a large number of highly mobile users. As a result, it is impossible to distribute orthogonal pilot sequences to every user in the network. Huge MIMO networks only have a few numbers of orthogonal pilot sequences available^28^. Therefore, we will emphasize non-orthogonal multi-access in MIMO networks and it’s pilot contamination attack.

##  The proposed detection scheme

The main idea of the proposed detection scheme is to detect the PCA attack in any BS or AS based on the abnormal behavior of its users. Our detection scheme is based on the signal power values as an indication of the user’s behavior variations.

**Fig. 3:**
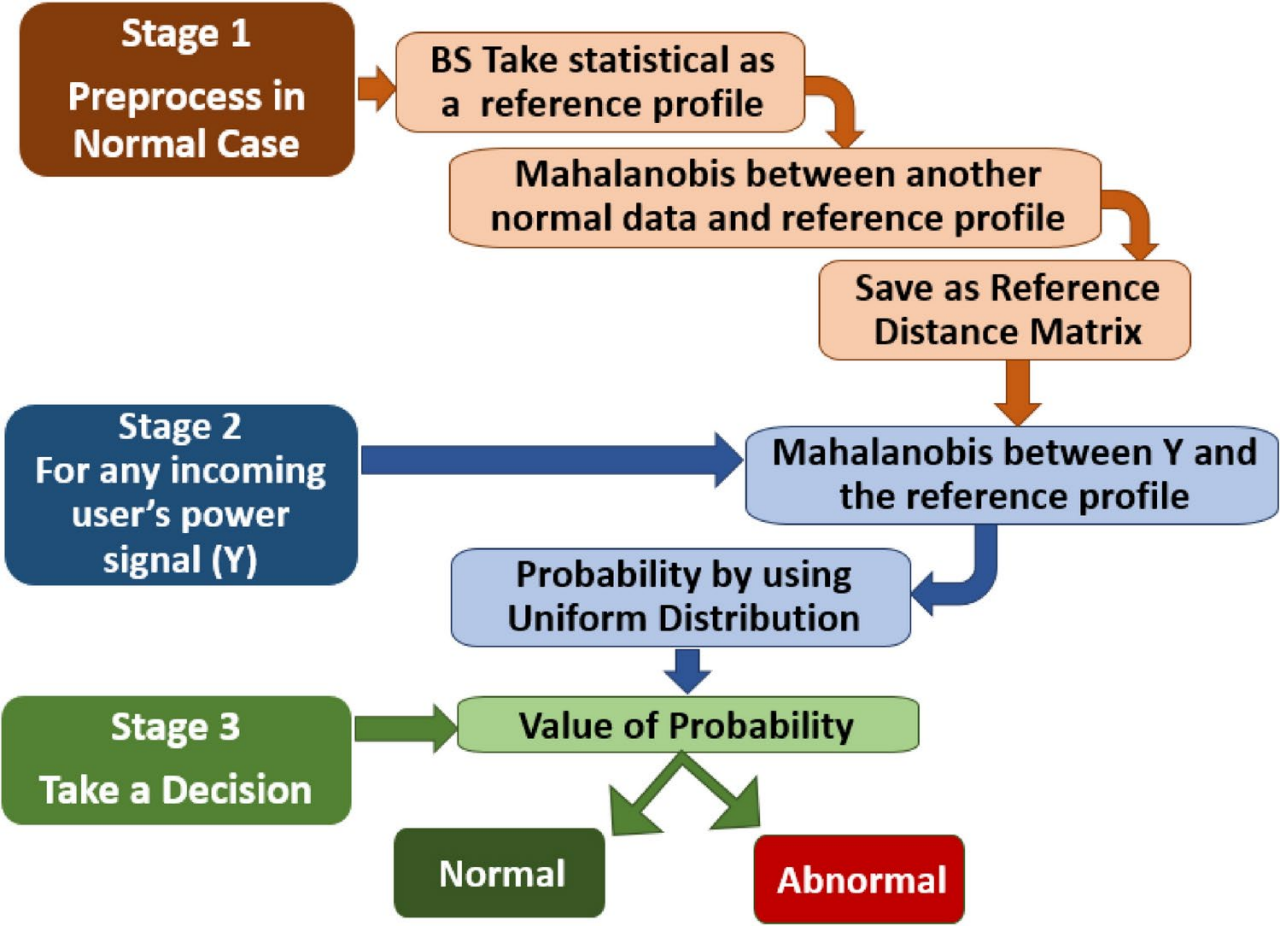
Diagram of proposed detection scheme stages.

The various signal power values during the normal time (i.e., no attacks) can be measured and saved by the BS to use later for the detection process. Thus, we apply the statistical method for quantifying the similarity between the previous values and any incoming user’s signal power to identify abnormal activity. Therefore, the proposed scheme can detect PCA with high sensitivity and precision. Fig. 3. illustrates the diagram for the three stages of our proposed detection scheme.

The proposed scheme can be explained in full detail using the Algorithm 1 based on the previous concept.

**Algorithm 1:**
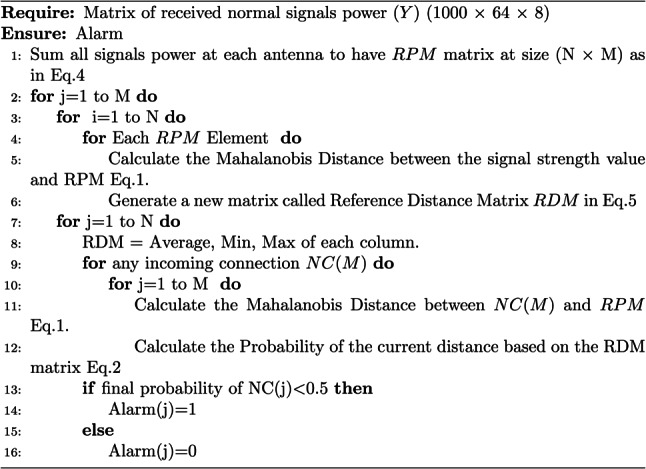
Detection of PCA using Mahalanobis distance

**Fig. 4:**
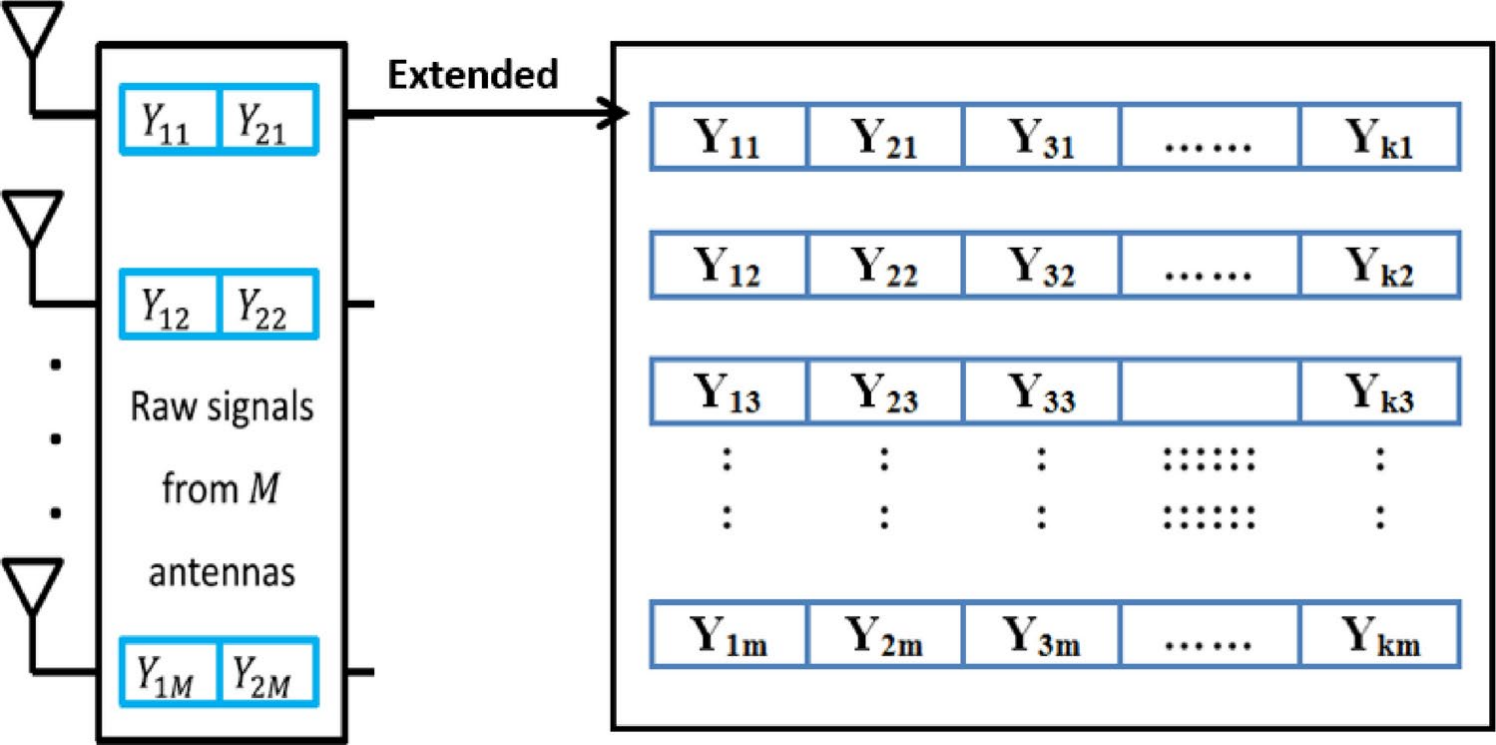
Reference profile matrix.

**Fig. 5:**
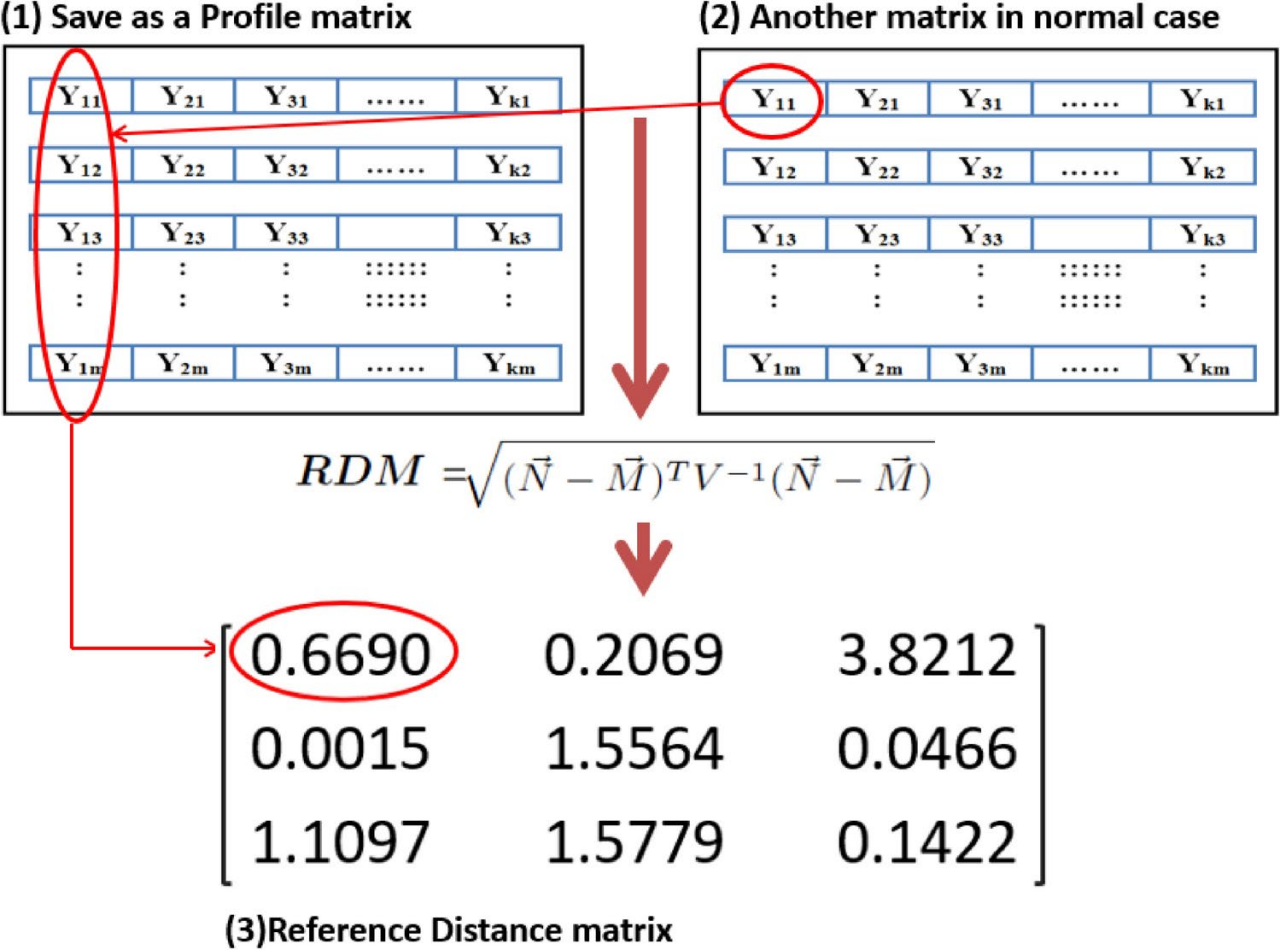
RDM calculation steps.

*The first stage* in our scheme is to measure signal strength values for all users of each antenna and keep these values as reference profile matrix as illustrated in Fig. 4. Then we compute the similarity between the previous reference profile and the incoming user’s signal power during normal time. The similarity is calculated by using the Multivariate Distances (i.e., Mahalanobis distance)^29,30^ between the signal strength value in the incoming normal traffic and the signal strength values in the reference profile as Eq. 1.

1 $$\begin{aligned} MD= \root \of {(\vec {N}-\vec {M})^{T} V^{-1}(\vec {N}-\vec {M})} \end{aligned}$$

where $\vec {N}$ is the value of any incoming signal strength during normal time and $\vec {M}$ is the vector of signal strength values in the reference profile and *V* is the covariance matrix of reference profile. By using Eq. 1, we obtain the Reference Distance Matrix (*RDM*) and used later in the stage 2 to compute the uniform model as shown in Fig. 5. Mahalanobis distance is used in the proposed detection scheme since it considers both individual variances and the covariance structure which makes it suitable for high dimensional data. This provides a robust measure of abnormal signal strength within the dataset.

*The second stage* is for any new incoming user’s signal power, the scheme calculates the Mahalanobis distance between the reference profile matrix and the incoming signal power. Then, the uniform distribution model Eq. 2 is used to calculate the probability for the current calculated distance based on the RDM matrix which is computed in stage 1. We use RDM for calculating the probability to know the minimum and maximum distance values.

2 $$\begin{aligned} \begin{aligned} F(z)= {\left\{ \begin{array}{ll} \frac{1}{(y-x)} , x \le z \le y \\ \\ 0 , O \cdot W \end{array}\right. } \end{aligned} \end{aligned}$$

*The third stage* Finally, our detection scheme makes a decision based on the previous probability value calculated in stage 2. If the probability is greater than 0.5, the incoming user is legitimate otherwise it is detected as the attacker.

## Performance evaluation

To evaluate the performance of the presented detection scheme, we conducted the simulation by using Matlab R2021a as^8^. This model simulate a single-cell massive MIMO system equipped with K single-antenna users and a M-antenna BS, so we can extend for antennas matrix to be as illustrated in Fig. 4. We used the correlated-Rayleigh fading channel to compute The channel vector ($h \in C^{M \times 1}$) between the BS antennae and each user same as the model in^31,32^:

3 $$\begin{aligned} h=R_{r}^{1/2}*h_{iid} \end{aligned}$$

where $R \in C^{M \times M}$ is the correlation matrix in the receiver side BS, which displays the correlation between antenna components in the BS, and $h_{iid}\in C^{M \times 1}$ is an independent realisation of a Rayleigh fading channel with Gaussian distribution.

The received signal $Y \in C^{M \times \tau }$ at the BS from all the authorized users is often stated as follows:

4 $$\begin{aligned} Y = \sum _{i=1}^{k}\sqrt{P_{i}}h_{i}\phi _{i}+n \end{aligned}$$

where $\sqrt{P_{i}}$ is the uplink transmit signal power of the *ith* user, $h_{i}\in C^{M \times 1}$ is the uplink channel of the *ith* user, and $\phi _{i} \in R^{1 \times \tau }$ is the non-orthogonal pilot sequence of *ith* user with length $\tau$. We used matrix with *K* non-orthogonal pilot sequences with length $\tau$ and $n\in C^{M \times \tau }$ is the additive white Gaussian noise matrix at the BS.

### Simulation setting

In this paper, we focus on PCA in uplink NOMA 5G communication. The BS in our system equipped with 64-antenna and each antenna have 8 legitimate users. Also, it use angular standard deviation in the local scattering model 10 degree, spacing between antennas is half the wavelength distance, the range of nominal angle of arrival $\Theta$ is 50 different value where $\Theta \in { [-\pi : \pi ] }$ and signal to noise ratio (SNR=0).

In the beginning of the simulation, all users send a pilot signal to BS, then BS estimates the CSI and retransmits it to all users. Thus, the communication channel is established between BS and all users. If there is any adversary needs to communicate with BS, it must copy the CSI of any legitimate users and uses it for communication. During attack scenario, each BS has 8 legitimate users and 8 adversary. Therefore, the signal power at each antenna will increase and this give a good indicator for attack detection.

In the presented detection method, we measure the distance between the signal power (*Y*) at each antenna and the covariance matrix during normal time to accurately detect the PCA attack. Thus the signal power at each antenna can be obtained by Eq. 5 as following:

5 $$\begin{aligned} Y = \sum _{i=1}^{k}\sqrt{P_{i}}h_{i}\phi _{i}+\sum _{a=1}^{k}\sqrt{P_{a}}h_{a}\phi _{a} + n \end{aligned}$$

where $\sqrt{P_{a}}$ is the uplink transmit power of adversary, (a = i = k).

**Fig. 6:**
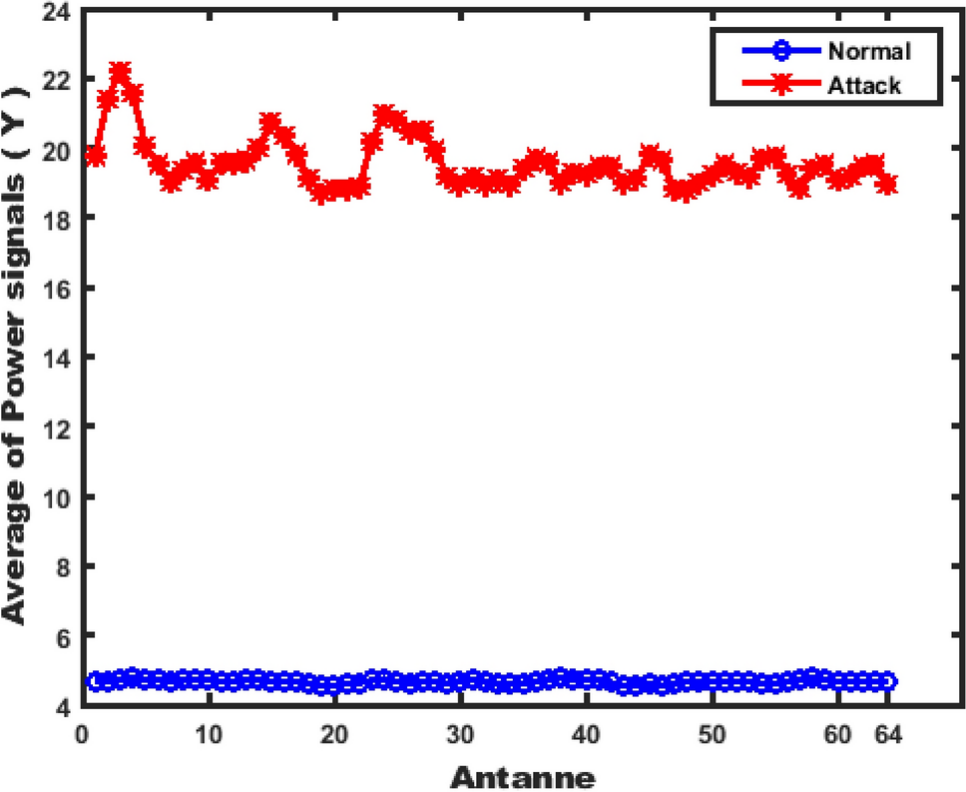
Antennas with Average of Power Signals at Each Antenna.

To clarify the increase in the signal power, we compute the average of signal power ($\bar{Y}$) in normal and attack scenarios as shown in Fig. 6. We can easily see from the figure that the signal power at attack time is higher than normal time. For example, in the normal case at antenna 3 the average signal power is 4.73 dB then it rises to 22.24 in the attack case. At antenna 24 the average signal power is 4.74 dB becomes 21.01 dB in the attack case. Finally, at antenna 38 the average signal power is 4.79 dB becomes 18.99 dB in the attack case.

### Experimental results

To study the performance of our proposed scheme under PCA attack, we conducted further simulation by using 1000 traces of normal & attack data and each trace has 64 antenna each with 8 users (i.e. 64 $\times$ 8 matrix for each trace). To evaluate the performance of the proposed scheme, we divided the traces of normal and attack traffic into 5 sub-traces as shown in Table 2. We examine the true positive, false positive, true negative and false negative probabilities for each sub-trace.

From Table 2 it is clear that, the proposed detection scheme succeeded to detect PCA attacks with a high detection rate, low false positives and low false negatives in normal and attack cases. For example, at normal case, the proposed scheme achieves a true negative value of 96.89 and a false positive of 3.1 for sub-trace 801:1000. At Attack case, the proposed scheme obtains a true positive value of 98.71 and a false negative of 1.28 at sub-trace 1:200.

Moreover, we compute the previous probabilities (i.e. true positive, false positive, true negative and false negative) for the total 1000 traces as shown in Table 2. The presented scheme can guarantee a true positive value of 98.29 and a false negative value 1.70 in attack case. On the other hand in normal case, achieves a true negative value of 97.86 and a false positive of 2.13

**Table 2:** The results of the proposed scheme.

Data traces	TP	TN	FP	FN
1:200	98.7109	96.5313	3.4688	1.2891
201:400	98.6719	96.7109	3.2891	1.3281
401:600	98.4141	95.9453	4.0547	1.5859
601:800	98.5313	96.0078	3.9922	1.4688
801:1000	98.6172	96.8906	3.1094	1.3828
Total	98.2953	97.8668	2.1332	1.7047

**Fig. 7:**
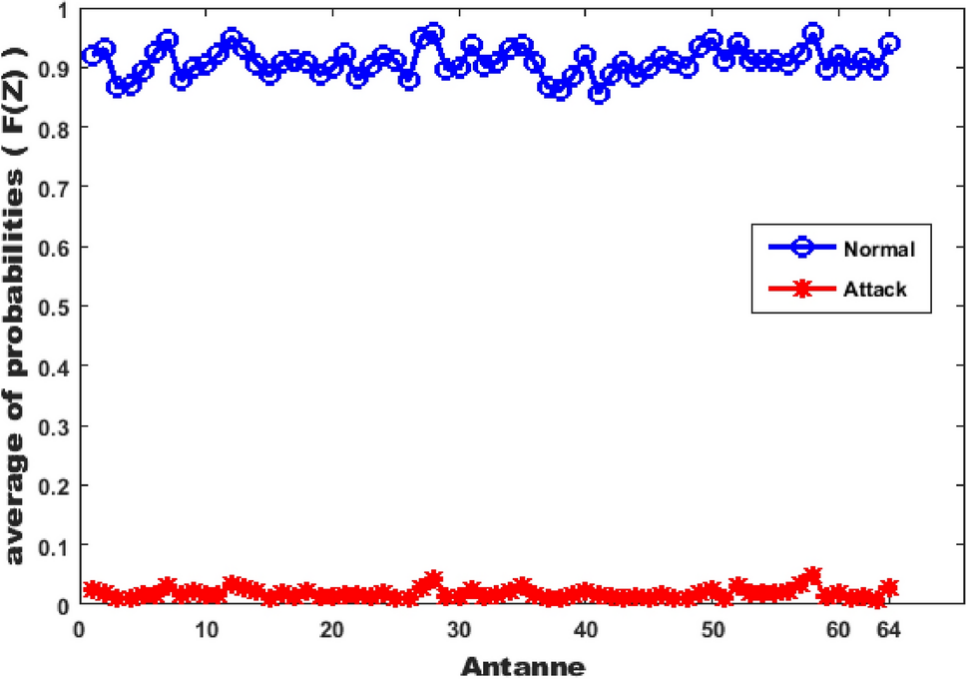
Antennas with average of probabilities at each antenna.

More evaluation can illustrated in Fig. 7 which is the relationship between each antennas and its average of uniform distribution probability values *F*(*z*). The uniform distribution probability is computed for distance between the signal power at each antenna and the Reference Profile Matrix. Fig. 7 shows that there is a high difference range between probabilities in normal and attack cases which make the proposed scheme sensitive for PCA attack. For example, at antenna 10 in the normal case, the average probability 0.904 decreased to 0.017 in the attack case. At antenna 28,the average probability 0.958 becomes 0.041 in the attack case. Finally, at antenna 60, the average probability 0.920 becomes 0.019 in the attack case.

To further study the performance of the proposed scheme, we examined the detection sensitivity of our scheme, Refs.^8,17,33^. The corresponding percentage of Accuracy, Precision, Recall, F1 Scoring of these detection methods are summarized in Table 3 respectively. It can be easily seen that our scheme outperforms other detection methods in Accuracy, F1 Scoring, Recall and Precision except for Ref.^8^. However, this difference in precision because^8^ used deep-learning algorithm to achieve 100% precision while our detection scheme used statistical model. It is notable that the proposed scheme achieves higher Accuracy, F1 Scoring and Recall than^8^.

**Table 3:** Comparison between proposed scheme with Refs.^8,33,17^.

Scheme	Accuracy	Precision	Recall	F1 Scoring
Proposed scheme	98.08	97.88	98.3	98.09
Ref.^8^	92	100	85.66	90.9
Ref.^33^	79.35	75.8	86.2	80.4
Ref.^17^	96.3	66.0	97.6	78.8

**Fig. 8:**
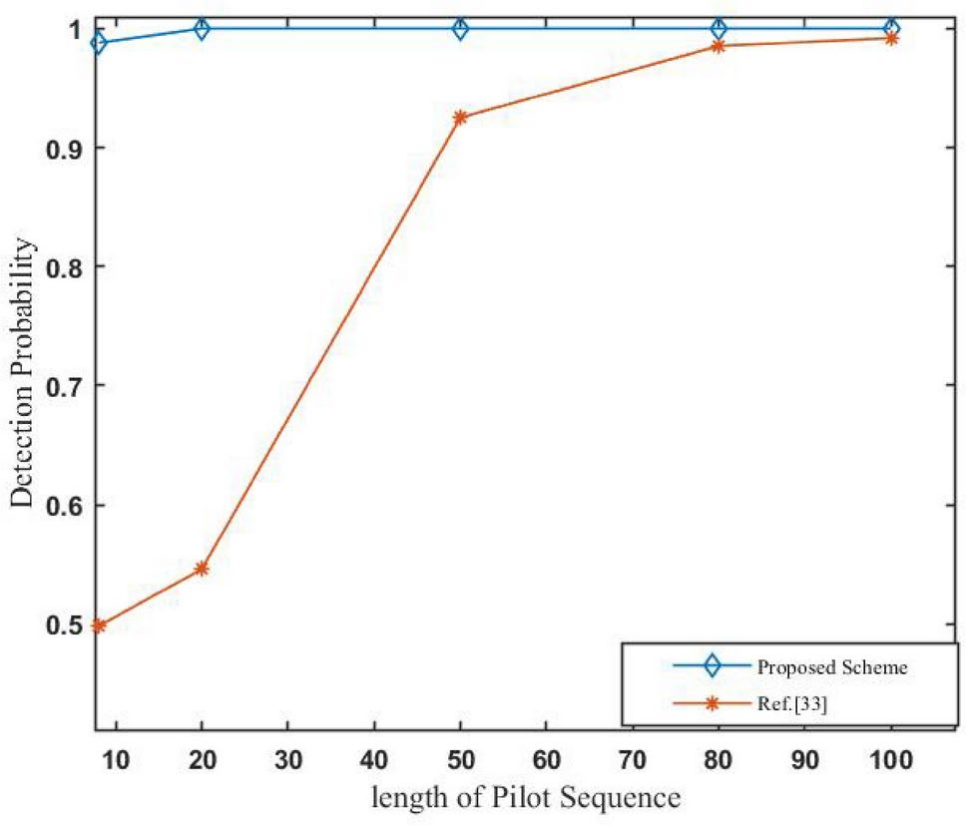
Detection Rates with different length of pilot sequence ($\tau$).

In Fig. 8, we show the detection probability with different lengths of pilot sequence to our scheme and scheme Ref.^33^ under the number of antenna=200 and various lengths of pilot sequence ($\tau$). From the figure, it can be seen that our scheme achieves significantly higher detection probability than Ref.^33^, especially with the increasing length of the pilot sequence. For example, when the $\tau$ = 8, the average detection probability of our scheme is more than 98% while the average detection probability of Ref.^33^ is just 49.8%. We can also easily see from the Fig. 8 that whenever the length of the pilot sequence 20, our scheme can guarantee 100% detection probability while the length of the pilot sequence must be above 100 for Ref.^33^ to get close to the same detection probability.

**Fig. 9:**
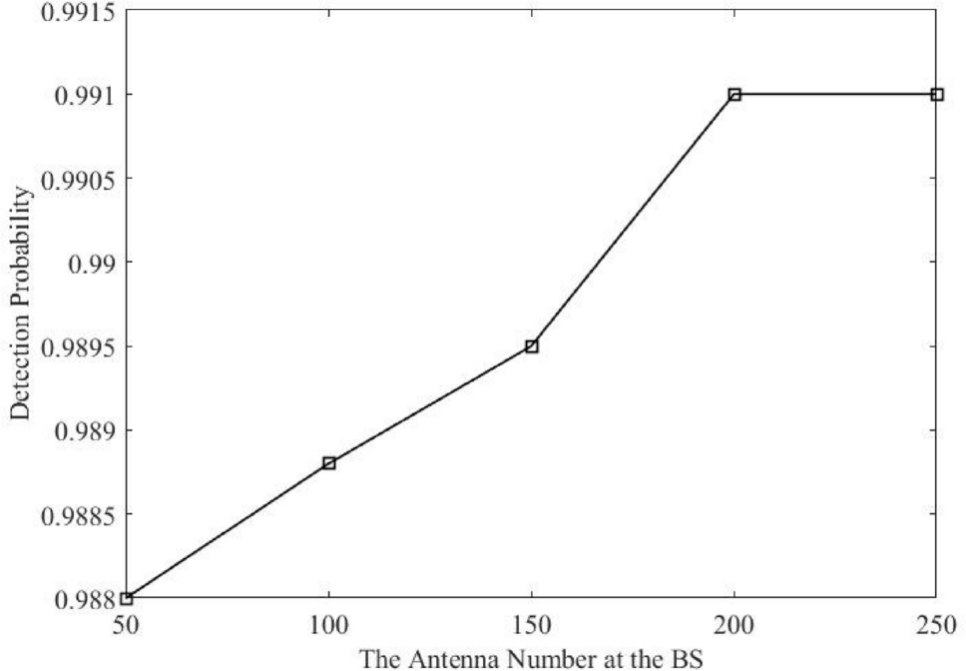
Detection rates with different number of antenna at BS.

The detection probability with the number of antennas (M) at BS was simulated and is displayed in Fig. 9 to illustrate the effect of increasing antenna number on the detection rate. It can be seen that improving the detection rate can benefit from having more antennas. For instance, with M = 50, 98.8 % of the potential pilot contamination attacks can be detected. Also, by using 200 antennas, the detection rate is increased to 99.1 %. Consequently, even with a small number of antenna, the proposed detection scheme performs well.

## Conclusion

This paper proposed detection scheme of PCA attack that enables the BS to detect the non-legitimate use of pilot sequences. Extensive experiments are demonstrated through computing score connection probability is presented by using Mahalanobis distance between a power signal for each antenna and statistical values in reference profile at normal case for BS to determine range of its normality. Due to its good sensitivity, the proposed scheme achieved detection rate of up to 98% and a precision reached 97.88%. While the proposed scheme demonstrates high detection results using simulation, we will verify its performance by mathematical modeling in the future work. Moreover, the presented detection method can be deployed on large scale 6G testbeds to validate its effectiveness in real world scenarios. Future research can also Combine traditional statistical detection methods with machine learning to introduce a robust attack detection.

## Data Availability

The datasets generated and analyzed during the current study are not publicly available since they are generated from our simulation program but are available from the corresponding author upon reasonable request.
